# The Golgi-localized, gamma ear-containing, ARF-binding (GGA) protein family alters alpha synuclein (α-syn) oligomerization and secretion

**DOI:** 10.18632/aging.101261

**Published:** 2017-07-15

**Authors:** Bjoern von Einem, Judith Eschbach, Martin Kiechle, Anke Wahler, Dietmar R. Thal, Pamela J. McLean, Jochen H. Weishaupt, Albert C. Ludolph, Christine A.F. von Arnim, Karin M. Danzer

**Affiliations:** ^1^ Department of Neurology, Ulm University, 89081 Ulm, Germany; ^2^ Laboratory for Neuropathology - Institute of Pathology, Ulm University, 89081 Ulm, Germany; ^3^ Department of Neuroscience, Mayo Clinic, Jacksonville, FL 32224, USA

**Keywords:** α-synuclein (α-syn), oligomers, golgi-localized, gamma ear-containing, ARF-binding (GGA), Parkinson's disease (PD), transport, secretion

## Abstract

Several age-related neurodegenerative disorders are associated with protein misfolding and aggregation of toxic peptides. α-synuclein (α-syn) aggregation and the resulting cytotoxicity is a hallmark of Parkinson's disease (PD) as well as dementia with Lewy bodies. Rising evidence points to oligomeric and pre-fibrillar forms as the pathogenic species, and oligomer secretion seems to be crucial for the spreading and progression of PD pathology. Recent studies implicate that dysfunctions in endolysosomal/autophagosomal pathways increase α-syn secretion. Mutation in the retromer-complex protein VPS35, which is involved in endosome to Golgi transport, was suggested to cause familial PD. GGA proteins regulate vesicular traffic between Golgi and endosomes and might work as antagonists for retromer complex mediated transport. To investigate the role of the GGAs in the α-syn oligomerization and/or secretion process we utilized protein-fragment complementation assays (PCA). We here demonstrate that GGAs alter α-syn oligomer secretion and α-syn oligomer-mediated toxicity. Specifically, we determined that GGA3 modifies extracellular α-syn species in an exosome-independent manner. Our data suggest that GGA3 drives α-syn oligomerization in endosomal compartments and thus facilitates α-syn oligomer secretion. Preventing the early events in α-syn oligomer release may be a novel approach to halt disease spreading in PD and other synucleinopathies.

## INTRODUCTION

Aging is the most prominent risk factor for a wide variety of neurodegenerative diseases. A common feature of these age-related diseases is protein misfolding and aggregation of toxic peptides due to age-related decline of cellular functions including protein sorting and degradation mechanisms. Parkinson's disease (PD), the second most common neurodegene-rative disorder, is characterized primarily by progressive degeneration of dopaminergic neurons in the substantia nigra pars compacta (SNpc). This neurodegeneration is accompanied by motor symptoms including tremor, bradykinesia, rigidity and postural instability. The major neuropathological hallmarks of PD are intraneuronal accumulations of misfolded protein, which are termed Lewy bodies (LB) and Lewy neurites (LN). The main component of LBs and LNs is aggregated alpha-synuclein (α-syn) [1]. Recent studies identified oligomeric intermediates of α-syn aggregates to be the predominantly neurotoxic species. Besides their toxic properties in cell culture experiments [12], α-syn oligomers are able to induce Parkinson-like symptoms in animal models [13]. Several factors, including oxidative stress [14], pH and temperature [10], post-translational modifications [15, 16], proteolysis [17, 18] and high concentrations of fatty acids [19–21], phospholipids and metal ions [14, 22, 23], were shown to induce and/or modulate α-syn structure and oligomerization *in vitro*. Emerging evidence suggests that α-syn oligomers spread from cell-to-cell and encourage the propagation of neurodegeneration in a prion-like manner [26]. In line with this, α-syn oligomers were found in conditioned media from cell cultures as well as human CSF and plasma [27–30]. It has been demonstrated that α-syn can be secreted from neuronal cells, enter other neighboring cells, and seed small intracellular aggregates [31]. We and others have shown that α-syn oligomers can be secreted in association with exosomes [32–35].

The physiological functions of α-syn are still a matter of debate but the current consensus is that monomeric α-syn is involved in the formation of synaptic vesicles and regulation of dopamine release [36]. Additionally, growing evidence indicates that α-syn is involved in the functioning of the neuronal Golgi apparatus and vesicle trafficking [37]. Disturbed vesicular transport is a common feature of neurodegenerative diseases. In this context, mutations in the gene of vacuolar protein sorting 35 (VPS35) were identified in inherited forms of PD [38–41]. The D620N mutation in VPS35 identified in familial PD cases leads to VPS35 loss of function which results in mis-sorting of cathepsin D, the protease responsible for α-syn degradation [45, 46] and impaired Lamp2a-mediated synuclein degradation [47].

A group of proteins likewise associated with the Golgi to endosome pathways are the Golgi-localized, gamma adaptin ear-containing, ARF-binding proteins (GGAs). The protein family consists of three members: GGA1, GGA2 and GGA3. GGAs are small adapter proteins recruited from the cytosol onto the Golgi where they mediate the transport of cargo [48–58] to endosomes/lysosomes [59, 60]. The n-terminal VHS (Vps27 (vacuolar protein sorting 27), Hrs (hepatocyte-growth-factor-receptor substrate), Stam (signal-transducing adaptor molecule)) domain binds cargo like the cation-independent Mannose-6-Phosphate Receptors (CI-MPR) and the cation-dependent MPR (CD-MPR) [48, 49, 52] and SorLA/LR11 [53], which are also transported by VPS35 [55, 61, 62]. GGA proteins can bind to rabaptin-5 a direct interactor of Rab5, which regulates endocytic transport at early endosomes [63–65]. Interestingly, an increase in toxicity was observed in Rab5A-specific endocytosis of α-syn in primary and immortalized neuronal cells [66]. However, whether GGAs directly bind to α-syn and alters its aggregation and/or secretion is not knowns to date.

We therefore addressed in this study the question of whether GGAs contribute to intracellular transport mechanisms involved in α-syn oligomerization and secretion leading to neuronal toxicity and eventually cell death.

## RESULTS

### Members of the GGA protein family alter α-synuclein oligomer secretion in cell culture

**Figure 1:**
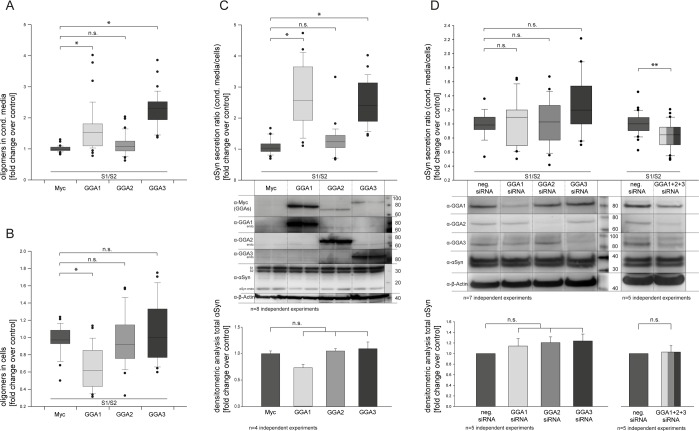
GGAs alter α-synuclein oligomer secretion in N2A cells N2A cells were co-transfected with α-syn fused to non-bioluminescent N- or C-terminal fragments of *Gaussia princeps* luciferase (α-syn-hGLuc1 (S1) and α-syn-hGLuc2 (S2)) and either empty control or one member of the GGA protein family. Then, 48 h after transfection, luciferase activity, indicative for α-syn oligomers, was determined in conditioned media as well as cells. GGA 1 and 3 co-transfected cells showed significantly higher luciferase activity in the conditioned media compared to controls (**A**), while intracellular levels of α-syn oligomers were not changed (GGA 2, 3) or even reduced (GGA1) (**B**). The ratio of secreted α-syn oligomers in the conditioned medium to intracellular α-syn oligomers was built showing increased secretion of α-syn oligomers for GGA1 and GGA3 (**C**). Experiments were carried out in triplicate; the results of n=8 independent experiments are shown. Densitometric analysis of corresponding Western blots revealed no significant difference in total α-syn levels upon expression of GGA2, 3 and the control. The mean fold change over control ±SEM of n=4 independent experiments is shown. Statistical analysis was performed using Kruskal-Wallis one-way analysis of variance (ANOVA) on ranks followed by multiple comparisons versus the control group (Dunn's Method) with *=p<0.05. The siRNA mediated knockdown of single endogenous Gga's abolished but did not reverse the effect of enhanced α-syn oligomer secretion in N2A cells (**D**). Experiments were carried out in triplicate; the results of n=7 independent experiments are shown. Statistical analysis was performed using Kruskal-Wallis one-way analysis of variance (ANOVA) on ranks (*=p<0.05). Simultaneous knockdown of all 3 Gga's significantly reduces α-syn oligomer secretion. Experiments were carried out in triplicate; the results of n=5 independent experiments are shown. Statistical analysis was performed using Mann-Whitney Rank Sum Test (**=p<0.008).

To address the question of whether members of the GGA protein family can alter α-synuclein oligo-merization and secretion, we used a protein-fragment complementation assay where α-syn was fused to non-bioluminescent N- or C-terminal fragments of *Gaussia princeps* luciferase (α-syn-hGLuc1 (S1) and α-syn-hGLuc2 (S2)) that reconstitute when brought together by α-syn/α-syn interactions [33, 67]. The constructs S1 and S2 were used to co-transfect N2A cells together with a myc-control plasmid or GGA1, 2 or 3, respectively. Cell lysates as well as conditioned media (CM) were analyzed 48h after transfection by measurement of luciferase activity indicative for α-syn oligomers. In contrast to GGA2, CM from GGA 1 and 3 co-transfected cells showed significantly higher luciferase activity compared to controls (Fig. 1A), while intracellular levels of α-syn oligomers were not changed (GGA 2, 3) or even reduced (GGA1) (Fig. 1B). To determine the secretion of α-syn oligomers, the ratio of α-syn oligomers in the CM and intracellular α-syn oligomers (luciferase CM divided by luciferase cells) was built (Fig. 1C). As demonstrated in Figure 1C, co-expression of S1, S2 and GGA1 or GGA3 resulted in a clear increase in the ratio of extracellular α-syn oligomers to intracellular α-syn oligomers. To control the equal expression of α-syn S1 and S2 Western Blots were performed (Fig. 1C) and quantified by densito-metric analysis (Fig. 1C, lower panel). We found similar α-syn S1/S2 expression levels indicating that increased α-syn oligomer secretion is not due to the increased expression of α-syn in the GGA 1 or GGA3 condition. To exclude cell line-specific effects, we additionally used HEK293 cells and over-expressed GGA1, 2 or 3 together with S1 and S2. As previously observed in N2A cells, the ratio of α-syn oligomers in the CM compared to intracellular α-syn oligomers of GGA1, 2 and 3 transfected cells was significantly higher as control transfected cells (Fig. S1). To address whether the increase in α-syn oligomers secretion was solely due to GGA proteins, a siRNA approach directed against GGA's was employed. Knockdown of single GGA's had no significant effect on α-syn oligomers secretion. However, simultaneous siRNA-mediated knockdown of all three endogenous GGA family members significantly reversed the effect of enhanced α-syn oligomer secretion via GGAs (Fig. 1D).

### GGA3 enhanced α-syn oligomer secretion is mediated mainly by exosome free pathways

**Figure 2:**
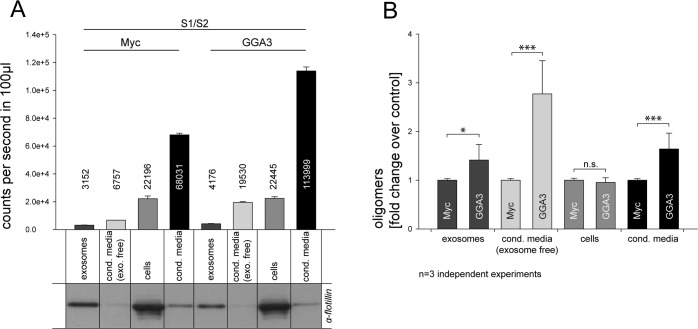
GGA3 enhanced α-syn oligomer secretion is mediated mainly by exosome-free pathways Conditioned media from N2A cells co-expressing S1/S2 and GGA3 or myc control were collected 48h after transfection. Exosomes were isolated from CM by subcellular fractionation. Purity of the fractions was confirmed by Western blot and the exosome-specific marker flotilin (**A**). As previously observed, α-syn oligomers, as measured by the luciferase activity, were increased in the conditioned media (**A,** lane4+8) but not cells (**A,** lane3+7) of GGA3 over-expressing cells compared to control. α-syn oligomers were detectable in exosomal fractions of both GGA3 over-expressing cells and controls with a small increase in exosomes (**A,** lane1+5) in the GGA3 condition but there was a higher increase in exosome depleted medium (**A,** lane2+6). Measurements were carried out in triplicate and the mean counts per second/100μl ±SEM of one representative experiment are shown. Normalization to myc control revealed a ∼3-fold increase of α-syn oligomers, as measured by the luciferase activity, in the exosome-free supernatant of GGA3 over-expressing cells (**B,** lane2+6) and only a ∼1.5- fold increase in the exosomal fraction (**B,** lane1+5). The mean fold change over the control ±SD of n=3 independent experiments is shown. Statistical analysis was performed by t-test with *=p<0.05, **=p<0.01,***=p<0.001.

Recent publications have shown that α-syn can be secreted to some extent via exosomes [33–35] but also exosome-independent pathways [32]. As GGA3 has shown the highest impact in secretion experiments, we used CM from N2A cells that were transfected with S1/S2 together with GGA3 or myc control plasmid to analyze by which pathway the observed increase in secretion is mediated. Exosomes were isolated from CM using an established subcellular fractionation metho-dology [68, 69]. To confirm the presence of exosomes, fractions from N2A cells were subjected to SDS-PAGE and immunoblotting. All exosomal fractions were found to be immunopositive for the exosome-specific protein flotillin, whereas the ‘exosome-free’ supernatant was immunonegative for flotillin (Fig. 2A). Luciferase activity was measured in cell lysates, non-purified CM, purified exosomes and exosome-free supernatants of GGA3 over-expressing cells and controls. As demonstrated in Figure 2A, α-syn oligomers were found in exosomal fractions of both GGA3 over-expressing cells and controls, with a small increase in exosomes in the GGA3 condition. However, a ∼3-fold increase in α-syn oligomers was found in the exosome-free supernatant in the GGA3 condition, whereas the increase in α-syn oligomers in the exosomal fraction was only ∼1.5-fold in the GGA3 condition compared to myc control (Fig. 2B). These results suggest that GGA3 mediates α-syn oligomer release mainly through an exosomal independent pathway.

### GGA3 and VPS4 have additive effects on α-syn oligomerization and secretion

**Figure 3:**
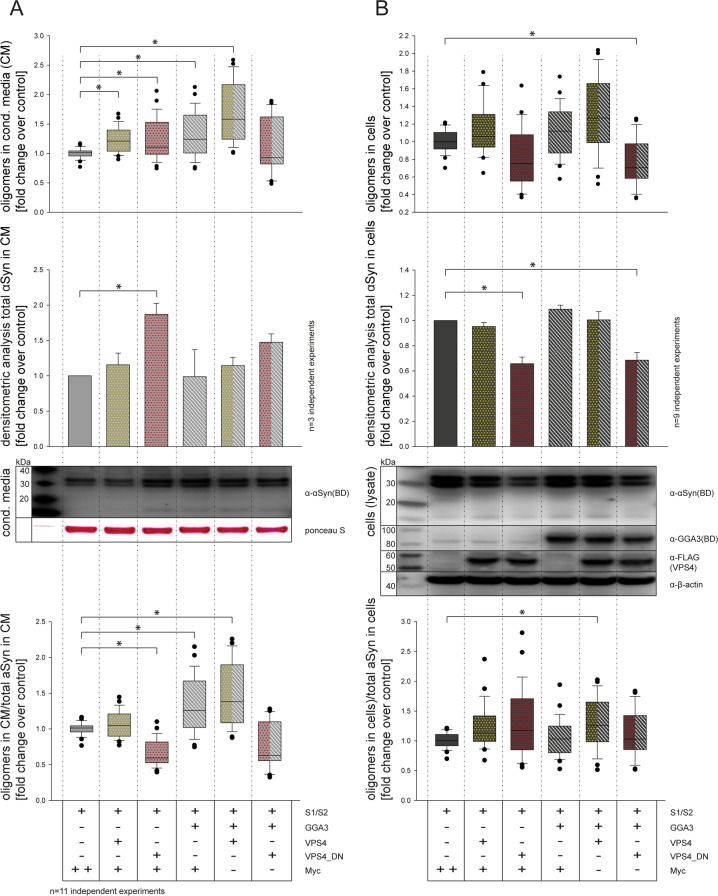
GGA3 and VPS4 have additive effects on α-syn oligomer secretion CM from N2A cells co-expressing S1/S2 and either empty control vector, GGA3, VPS4 and VPS4_dn alone or in combination were collected 48h after transfection. α-syn oligomer levels were measured by luciferase assay in conditioned media (CM) (**A**) as well as cells (**B**). The upper panel of (**A**) shows α-syn oligomer levels in CM measured by luciferase assay as fold change to control (Myc). The middle panel of (**A**) shows the total -syn levels in CM measured by densitometric analysis as fold change to control. The lower panel of (**A**) shows the proportion of a-syn oligomers in CM to total α‐syn in CM as fold change to control. The upper panel of (**B**) shows α‐syn oligomer levels in cells measured by luciferase assay as fold change to control (Myc). The middle panel of (**B**) shows the total α‐syn levels in cells measured by densitometric analysis as fold change to control. The lower panel of (**B**) shows the proportion of α‐syn oligomers in cells to total α‐syn in cells as fold change to control. In proportion to total α‐syn, VPS4 and GGA3 overexpression increased α‐syn oligomer secretion (**A,** lower panel, lane 4+5). In contrast, expression of VPS4_dn resulted in decreased α‐syn oligomerization in CM (**A,** lower panel, lane 3), whereas intracellular α‐syn oligomer as well as total α‐syn levels were decreased (**B,** upper and middle panel, lane 3+6). The results of n=11 independent experiments are shown. Statistical analysis was performed using Kruskal‐Wallis one‐way ANOVA on ranks followed by multiple comparisons versus control group (Dunn's Method) with *=p<0.05.

To further analyze the pathway involved in GGA3-mediated α-syn oligomer secretion, we tested a possible additive effect of GGA3 and VPS4 or dominant-negative effect of VPS4 (VPS4_dn) and GGA3, respectively. VPS4 is required in multivesicular body (MVB) biogenesis and intraluminal vesicle (ILV) formation and thereby a regulator of exosome formation [70]. Furthermore, it was previously described that blocking of the MVB pathway by expression of a dominant-negative VPS4 form increases α-syn secretion via recycling endosomes [32]. As measured previously, the expression of GGA3 in N2A cells increased the levels of α-syn oligomers in the conditioned media compared to control as measured by luciferase assay (Fig. 3A upper panel, lane 1+4) without altering the level of total α-syn in the conditioned media as measured by Western blot analysis (Fig. 3A middle panel, lane 1+4). The expression of VPS4 also slightly increased levels of α-syn oligomers in the conditioned media (Fig. 3A, upper panel, lane 2). The amount of total α-syn in the conditioned media was not changed upon VPS4 expression (Fig. 3A middle panel, lane 2). For VPS4_dn on the other hand, we found a significant increase in total α-syn in the conditioned media compared to control (Fig. 3A middle panel, lane 3). The co-expression of GGA3 and VPS4 further increased the level of α-syn oligomers in the conditioned media without altering total α-syn levels (Fig. 3A upper and middle panel, lane 5). Co-expression of GGA3 and VPS4_dn on the other hand showed non-significant but increased levels of total α-syn in the conditioned media without altering level of α-syn oligomers in the conditioned media (Fig. 3A upper and middle panel, lane 6). To get an impression of the oligomerization rate for each condition, we calculated the proportion of oligomers to total α-syn in conditioned media compared to control (Fig. 3A lower panel). Compared to control, we found a significant decrease in oligomerization for VPS4_dn expressing cells (Fig. 3A lower panel, lane 3), whereas expression of GGA3 and co-expression of GGA3 and VPS4 increased oligomer/total α-syn levels in the conditioned media (Fig. 3A lower panel, lane 4+5). We performed the same procedure for the intracellular α-syn levels (Fig. 3B). Although not significantly, expression of VPS4_dn alone slightly decreased α-syn oligomer levels in the cells (Fig. 3B upper panel, lane 3). Co-expression of GGA3 and VPS4_dn let to a significantly decreased level of intracellular α-syn oligomers (Fig. 3B upper panel, lane 6), whereas all other conditions where similar compared to control (Fig. 3B upper panel, lane 1-2, 4-5). Western blot analysis revealed significantly reduced intracellular levels of total α-syn in cells expressing VPS4_dn (Fig. 3B middle panel, lane 1-6). Similarly, to the conditioned media, we calculated the proportion of intracellular oligomers to intracellular total α-syn compared to control (Fig. 3B lower panel). After correction to total α-syn, only co-expression of GGA3 and VPS4 showed a significant increase in oligomerization compared to control (Fig. 3B lower panel, lane 5). Taken together, we found increased total α-syn levels but decreased α-syn oligomer levels in the conditioned media of cells overexpressing VPS4_dn. Furthermore, we found reduced intracellular levels of both total α-syn and α-syn oligomers upon VPS4_dn expression. These findings might be explained by an accelerated secretion of α-syn through pathways that are not involved in oligomerization. The accelerated secretion of α-syn via recycling endosomes for example, has been described previously [32]. Expression of GGA3 or VPS4 and co-expression of both proteins slightly but not significantly increased oligomer levels intracellularly without altering the amount of total α-syn significantly. However, correction to total α-syn levels indicate that co-expression of GGA3 and VPS4 had an additive effect on α-syn oligomerization and secretion. Taken into account that both, GGA3 as well as VPS4, promote transport into the late endosomal/MVB pathway, it is reasonable to speculate that expression of both proteins might enhance α-syn oligomerization in these compartments.

### GGA modifies secreted α-syn oligomer species and exacerbates extracellular α-syn toxicity

**Figure 4:**
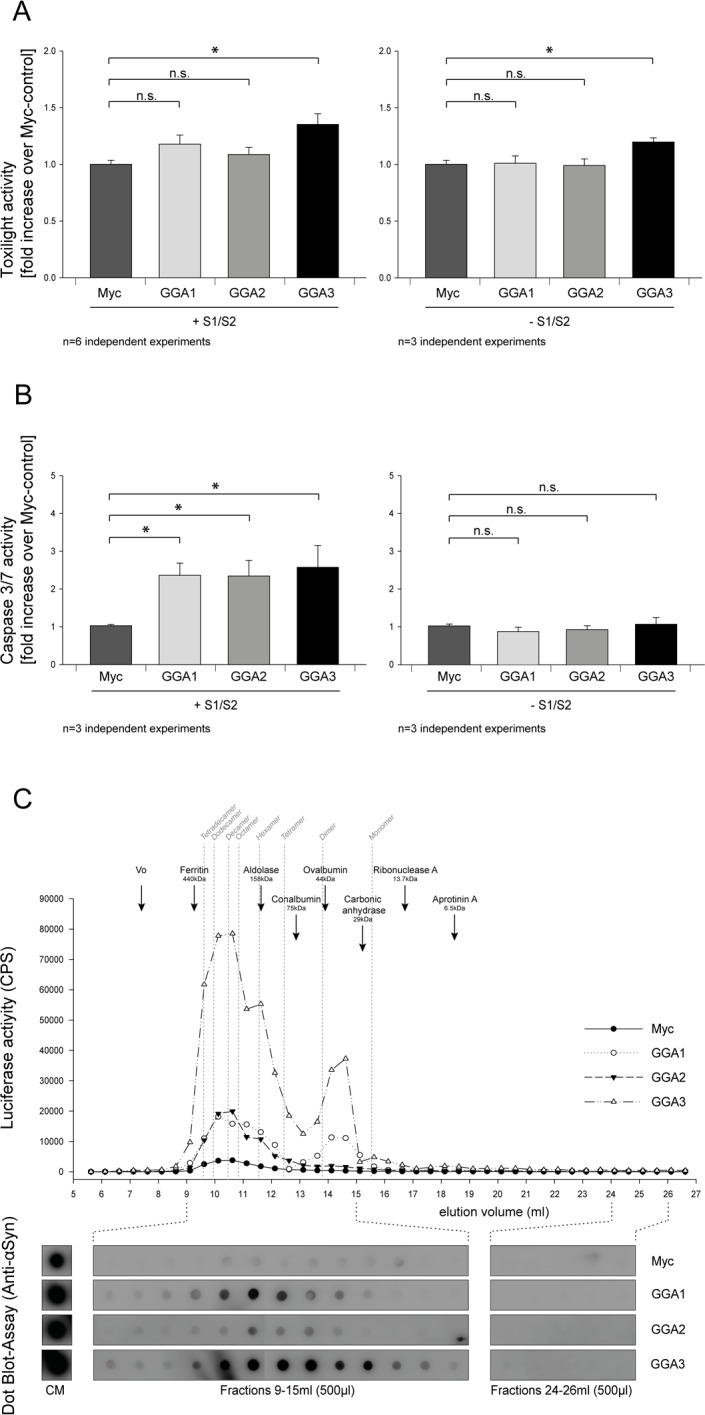
GGA modifies secreted α-syn oligomer species and exacerbates extracellular α-syn toxicity GGA1, 2, 3 or myc control were expressed alone or with S1 and S2 in N2A cells. Then, 48h after transfection, conditioned media were collected and analyzed for increased cytotoxicity using the ToxiLight assay (Lonza). Neither GGA1 nor GGA2 co-expression with S1 and S2 showed enhanced toxicity compared to myc-control whereas a slight increase was observed upon GGA3 expression (**A**, left panel). The mean fold change over control ±SEM of n=6 independent experiments is shown. To test for the GGA-dependent increase in cytotoxicity, GGA1, 2, 3 or myc control were expressed without S1/S2 in N2A cells. GGA3 expression slightly increased toxicity compared to control, GGA1 and GGA2 (**A**, right panel). The mean fold change over control ±SEM of n=3 independent experiments is shown. Statistical analysis was performed using Kruskal-Wallis one-way ANOVA on ranks followed by multiple comparisons versus control group (Dunn's Method) with *=p<0.05. CM from cells expressing GGA1, 2, 3 or myc-control alone or with S1 and S2 was transferred to naïve N2A cells. After 72h, cells were analyzed for altered Caspase 3/7 activity. We found that CM from N2A cells co-expressing S1/S2 together with GGA1, 2 and 3 caused a 2-fold increase in Caspase 3/7 activity on naïve N2A cells compared to CM from N2A cells that were co-transfected with S1/S2 together myc-control plasmids (**B**, left panel). In contrast, CM from N2A overexpressing GGA1, 2, 3 or myc control alone had no influence on Caspase 3/7 activity (**B**, right panel). These findings indicate that GGA not only alters the amount of secreted α-synuclein oligomers but also the quality of oligomeric species in the conditioned media. The mean fold change over control ±SEM of n=3 independent experiments is shown. Statistical analysis was performed using Kruskal-Wallis one-way ANOVA on ranks followed by multiple comparisons versus control group (Dunn's Method) with *=p<0.05. CM from HEK293 cells co-expressing S1/S2 and GGA3 or myc-control was analyzed by size exclusion chromatography (SEC). Indicated in grey are the calculated sizes of different α-syn oligomers and their estimated elution volumes. Fractions of 0.5 ml were collected and further analyzed for α-syn by luciferase assay and Dot Blots. We detected a heterogeneous population of oligomeric α-syn in CM of S1/S2 transfected cells secreted from cells ranging from multimers to dimers (**C**). GGA co-expression increased extracellular α-syn dimers but also multimers. These results support the idea that co-expression of GGAs change α-syn oligomer formation, species and secretion.

To further characterize the influence of GGAs on α-syn oligomer secretion we asked whether the co-expression of S1/S2 together with GGA protein family members can enhance cytotoxicity of α-syn oligomers (S1/S2). The Toxilight assay measures the release of adenylate kinase into the conditioned media. Therefore, this assay can be used as an indicator for unspecific release of proteins from the cytoplasm. As demonstrated in Figure 4A no overt toxicity was seen upon co-expression of S1, S2 and GGA1 or GGA2 in N2A cells compared to the myc-control plasmid. A small increase in cytotoxicity was observed when S1/S2 was co-expressed with GGA3 (Fig. 4A, left panel). As a control, we also determined the toxicity of cells expressing GGAs alone (Fig. 4A, right panel). Toxicity in cells overexpressing GGA3 alone was slightly increased compared to the control. This indicates that increased toxicity in cells overexpressing S1 and S2 together with GGA3 is likely due to GGA3 expression.

We, and others, have previously shown that the exogenous addition of α-syn oligomers can induce Lewy-body-like pathology and related toxicities [12] [31] [71]. Cell-to-cell transmission of pathological α-syn and spreading of α-syn pathology has been demonstrated in a number of studies [72–74]. To determine whether CM from cells that were co-transfected with S1/S2 together with GGA1, 2 and 3 is more toxic to naïve cells compared to those from cells co-expressing S1/S2 and myc-control, we added CM from S1/S2 co-transfected N2A cells with GGA1, 2, 3 or myc control plasmid to naïve N2A cells. To ensure that equal amounts of α-syn oligomers were applied to naïve cells, equal amounts of luciferase counts were used for each treatment. After 72h, N2A cells that were incubated with different CMs were analyzed for Caspase 3/7 activity. We found that CM from N2A cells that were co-transfected with S1, S2 and GGA1, 2 or 3 showed a 2-fold increase in Caspase 3/7 activity compared to CM from N2A cells that were co-transfected with S1, S2 and myc-control plasmids (Fig. 4B, left panel). In contrast, neither CM from cells transfected with GGA1, 2 nor 3 alone was able to increase toxicity in naïve cells compared to control (Fig. 4B, right panel). These findings indicate that GGA not only alters the amount of secreted α-syn oligomers but might also change the profile of the heterogeneous population of different oligomer species in the conditioned media.

We have seen before that the majority of α-syn oligomers are secreted by exosome-free pathways. However, it is not clear whether the α-syn species responsible for the increased toxicity are transported by exosomes or exosome-free pathways. To address this question naïve N2A cells were treated with exosome-loaded and exosome-free media of N2As co-expressing S1, S2 and GGA3 or myc-control. We found a significantly increased Caspase3/7 activity in receptor cells incubated with GGA3-exosomes compared to those incubated with myc-exosomes (Fig. S2, upper panel) whereas no difference in the toxicity of the exosome-free samples was observed. In addition, we observed a trend towards an increased release of adenylate kinase as measured by the ToxiLight assay from those cells incubated with GGA3-exosomes compared to myc-exosomes. No difference was observed between the exosome-free conditions (Fig. S2, lower panel). These data suggest that, although a lower percentage of total α-syn oligomers are secreted by the exosome pathway than by the exosome-free pathways, the more toxic α-syn species are secreted via exosomes. To further characterize the released α-syn species in the CM from cells expressing S1/S2 together with GGAs or myc-control, we applied size exclusion chromatography (SEC). We took advantage of the luciferase complement assay and performed a luciferase assay on all fractions from S1/S2/myc and S1/S2/GGAs CM to detect oligo-meric α-syn. We detected a heterogeneous population of oligomeric α-syn in CM of S1/S2 transfected cells secreted from cells ranging from ∼30mers to dimers (Fig. 4C). Co-transfection with GGAs increased dimers as well as species found between 9 and 12 ml in SEC fractions of CM of HEK293 cells (Fig. 4C). These results imply that the co-expression of GGAs enhances oligomerization and alters the composition of α-syn species in CM.

### GGA3 is expressed in the substantia nigra

**Figure 5:**
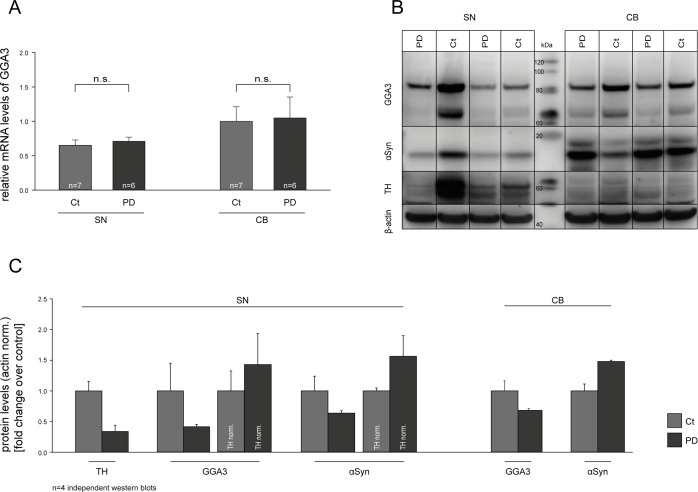
GGA3 is expressed in the substantia nigra Real-time quantitative PCR (RT-qPCR) was performed on human post-mortem substantia nigra (SN) and cerebellum (CB) samples of PD patients and non-PD controls. mRNA levels of GGA3 were not altered in PD patients compared to controls (**A**). Western blot analysis of postmortem samples showed decreased GGA3 and α-syn protein levels in SN but not CB samples of PD patients compared to controls **(B)**. A representative Western blot of n=4 blots is shown. Densitometric analysis and normalization to tyrosine hydroxylase (TH) levels indicate that reduction in GGA3 levels are due to loss of dopaminergic neurons in the SN of PD patients **(C)**. PD protein levels of each blot were normalized to the non-PD protein levels of the corresponding blot. The mean fold changes over control ±SEM of n=4 blots is shown.

Decreased levels of GGA1 and GGA3 have been described for Alzheimer's disease [75–77]. However, to our knowledge no data are available for GGAs and PD. Based on our findings, we addressed the question whether GGA3 might be deregulated in PD on RNA and/or protein level and thereby influence α-syn aggregation and secretion and PD pathology. Real-time quantitative PCR (RT-qPCR) was performed on human post-mortem samples from the substantia nigra (SN) and cerebellum (CB) of PD patients and non-PD controls. The characteristics of the patients from which the post mortem tissue samples have been obtained are listed in supplementary table 1 (Tab.S1). We found no altered mRNA levels of GGA3 in PD patients compared to controls (Fig. 5A). Furthermore, we performed Western blot analysis with the same postmortem samples. GGA3 protein levels were decreased in SN samples of PD patients compared to controls, whereas no difference was observed in CB (Fig. 5B). However, normalized to tyrosine hydroxylase (TH) levels, reductions in GGA3 levels are likely due to the loss of dopaminergic neurons in the SN of PD patients (Fig. 5C).

## DISCUSSION

The current consensus on how α-syn contributes to the development of Parkinson's disease includes misfolding of the protein, aggregation into toxic species, spreading of these toxic species throughout the brain and seeding of aggregation in a prion-like manner. Since oligomeric intermediates have been identified to be the neurotoxic species, research has focused on the mechanisms and transport pathways involved in the assembly and secretion of α-syn oligomers. Besides the fact that α-syn itself was found to participate in vesicle transport at endosomal and Golgi compartments [78], recent publications identified mutations in VPS35 in familial PD [38–41]. Here, we provide evidence that the GGA protein family contributes to the aggregation and secretion of α-syn oligomers.

Using protein complementation assays, we found increased levels of α-syn oligomers in CM of N2A as well as HEK293 cells over-expressing GGA1, 2 or 3 (Fig. 1A-C+Fig. S1), which could be reversed by siRNA mediated knock-down of all three GGAs simultaneously (Fig. 1D). All three human GGAs display high homology and participate in transport and sorting of the same cargo in the same pathway [59]. Therefore, a compensatory effect upon knock-down of one GGA is conceivable and has been observed previously [79]. In line with this, deletion of Yeast-GGA1 or Yeast-GGA2 alone had no or only minor effects, whereas deletion of both genes causes notable trafficking defects in distinct post-Golgi events [80–83]. However, upon over-expression of GGAs, intracellular α-syn total levels are not altered, as shown by Western blot analysis (Fig. 1+Fig. S1). This indicates that increased levels of α-syn oligomers in the conditioned media of cells over-expressing GGAs are not due to altered α-syn expression but altered transport and enhanced oligomer secretion.

Concerning α-syn secretion, the involvement of different pathways has been suggested. Recently, it was reported that α-syn is secreted by a pathway involving early endosomal and recycling compartments [32]. On the other hand, we and others have previously reported that α-syn can be secreted via exosomes [33, 35]. Additionally, exosome-mediated cell-to-cell transmission of α-syn oligomers has also been demonstrated [33]. Western blot analysis following exosome purification revealed that α-syn oligomers are mainly increased in exosome-free fractions of cells over-expressing GGA3 (Fig. 2). However, it does not show that secretion of α-syn oligomers is independent of late endosomes/MVBs. Furthermore, the increase in toxicity mediated by conditioned media of cells over expressing S1, S2 and GGA3 is associated mainly to the exosomal fraction (Fig. S2). As GGAs are known to transport cargo into the endolysosomal pathway it is likely that GGA mediated alterations of α-syn their over-expression drives vesicular transport of also occur in this pathway. Finally, co-expression of GGA3 and VPS4 but not VPS4_dn had an additive effect on α-syn oligomer secretion but did not alter intracellular total α-syn levels (Fig. 3). In these experiments, the impact of GGA3 alone on α-syn oligomer secretion is weaker than that observed in previous experiments (Fig. 1). For these experiments, we performed co-transfection with 4 different expression vectors and therefore reduced the amount of S1/S2 as well as GGA3 compared to the previous experiments. The lowered plasmid con-centrations directly affect expression of each protein and thereby the secretion of α-syn oligomers. It has been reported previously, that expression of dominant negative VPS4 enhances total α-syn secretion via early endosomal and recycling pathways [32], whereas α-syn oligomer secretion has not been addressed discretely in this report. In line with this, levels of total α-syn were increased in the CM of cells co-expressing S1/S2 and VPS4_dn while intracellular levels were decreased as seen by Western blot. α-syn oligomer levels were also slightly increased in CM, but proportionally to total α-syn levels in CM the α-syn oligomerization was reduced. Furthermore, we found decreased intracellular α-syn oligomer and total α-syn levels upon VPS4_dn expression alone or with co-expression of GGA3., Co-expression of VPS4 alone non-significantly and in combination with GGA3 significantly enhanced intra-, as well as extracellular levels of α-syn oligomers. These data might indicate that blocking of the endolysosomal pathway increases monomeric α-syn secretion via early- and recycling endosomes. In contrast, α-syn oligomerization and oligomer secretion is enhanced in VPS4 and GGA3 pathways. Therefore, we speculate that GGA3 contributes to the enhanced transport of α-syn into or increased residence of α-syn in the endolysosomal pathway which leads to enhanced α-syn oligomerization in these compartments.

It has been previously described that GGAs interact with rabaptin-5 a direct effector of the early endosomal small GTPase Rab5 [63]. Furthermore, α-syn toxicity was reported to be increased upon Rab5 dependent endocytosis [66]. We therefore speculate that GGAs contribution to the transport to endosomes promotes α-syn toxicity through enhanced oligomerization. Additional support for this idea comes from recent findings. Follett and colleagues have shown that the VPS35 D620N mutation linked to PD disrupts the cargo sorting function of the retromer complex [45]. They found enlarged early endosomes and mis-sorting of cathepsin D that is responsible for α-syn proteolysis in the lysosomal compartments [84, 85]. Taken into account all findings, it is rather unlikely that the GGA3 and VPS4 effect on α-syn are directly interconnected. However, the exact mechanism of the additive effect on α-syn oligomerization and secretion upon co-expression of both proteins has to be addressed in future studies. To test whether GGA-mediated transport contributes to enhanced oligomerization, we applied SEC and subsequent luciferase measurements of conditioned media. We found an increase in oligomeric species of higher molecular weight (Fig. 4C) upon co-expression of GGAs. These findings further support the idea that transport through endosomal compartments mediated by GGAs do enhance α-syn oligomerization. Furthermore, the shift in α-syn oligomer species due to GGA3 co-expression was accompanied by an increased toxicity when CM of cells over-expressing GGAs were applied to naïve cells (Fig. 4B). We thus speculate that GGAs mediate α-syn transport to endosomal compartments leading to increased oligomer generation with subsequent increased α-syn oligomer secretion.

**Figure 6:**
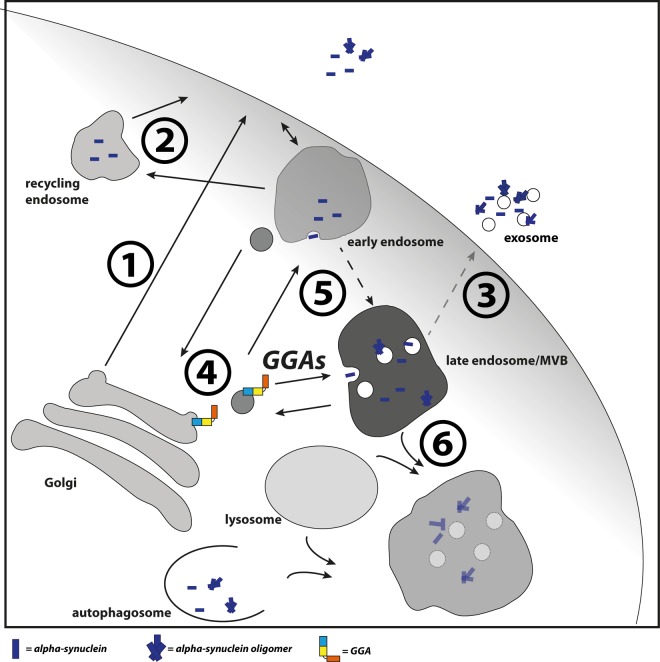
GGAs contribute to α‐syn transport into the endolysosomal pathway Different pathways have been described which contribute to α‐syn secretion. This includes the secretory pathway **(1)**, recycling pathway via early endosomes and recycling endosomes **(2)** and upon disturbed lysosomal degradation the release of MVB content at the plasma membrane **(3)**. GGAs contribute to α‐syn transport or occurrence of α‐syn in the endolysosomal pathway. Intracellular α‐syn aggregation and secretion can be triggered by different mechanisms. The D620N mutation in VPS35 identified in familial PD cases lead to VPS35 loss of function and mis‐sorting of cathepsin D **(4)** and thereby to reduced lysosomal degradation of α‐syn. GGAs might increase α‐syn aggregation directly through enhanced transport into acidic environment or indirectly through enhanced residence in the acidic pH **(5)**. In case of disturbed lysosomal function **(6)**, α‐ syn oligomer loaded MVBs will fuse with the plasma membrane and release their content into the extracellular space **(3)**.

It is unclear which mechanism underlies enhanced oligomerization in endosomes and how GGAs contribute to this event. It is possible that enhanced transport and thereby enhanced occurrence of α-syn in these compartments trigger oligomerization. Early endosomes become increasingly acidic during maturation to late endosomes/MVBs and it is known that α-syn aggregates more rapidly under these conditions [86]. Additionally, CI-M6PR is a prominent cargo of GGAs and is transported by these from TGN to endosomes [48, 49, 52]. As recently reported, CI-M6PR is the receptor for cathepsin D at the TGN, a protease involved in lysosomal degradation of α-syn. Disturbed transport of CI-M6PR from early endosomes to TGN caused by disturbed retromer function resulted in accumulation and secretion of immature pro-cathepsin D [45]. Likewise, it is possible that over-expression of GGAs enhances the retention of pro-cathepsin D at the TGN through increased CI-M6PR occurrence in endosomes. This might lead to reduced lysosomal degradation of α-syn and enhanced oligomerization and secretion via the fusion of MVBs with the plasma membrane. Though we have not seen interaction of GGA3 and α-syn in co-immunoprecipitation experiments (Fig. S3), we do not want to exclude the possibility of GGA mediated transport of α-syn. However, we suggest a model in which GGAs contribute to α-syn transport or occurrence in the endolysosomal pathway (Fig. 6). In the case of disturbed lysosomal degradation, as found in PD, GGAs might increase α-syn aggregation directly through enhanced transport into an acidic environment or indirectly through enhanced residence in the acidic pH.

Finding GGA3 and α-syn co-expressed in dopaminergic neurons of human substantia nigra samples provides hints that the observed effects might also occur *in vivo*. Since only samples of two control and two PD patients could be included in the study statistics could not be performed and we can only speculate regarding the role of GGA3 dysregulation in the human situation. However, we found no alterations in GGA expression in the PD cases analyzed in this study indicating that further research is needed to fully understand the role of GGAs in the human brain.

Taken together, we here provide evidence that the GGA protein family can alter α-synuclein transport, oligomerization and secretion. This study sheds light on the mechanisms involved in α-syn toxicity found in Parkinson's disease and other alpha-synucleinopathies. To understand the pathways involved in oligomer secretion and spreading of neurodegeneration in the brain will offer new therapeutic targets in the attempt to develop promising drugs to treat Parkinson's disease.

## MATERIALS AND METHODS

### Antibodies and expression constructs

The following primary antibodies were utilized: mouse (ms) α α-synuclein (α-syn) (BD/610787), rabbit (rb) α GGA1 (H215, Santa Cruz/sc-30102), ms α GGA2 (BD/612612), ms α GGA3 (BD/612310), ms α c-myc (9E10, Sigma-Aldrich/M4439), ms α β-actin (AC-15, Sigma-Aldrich/A5441), rb α FLAG (Sigma-Aldrich/F7425), rb α tyrosine hydroxylase (TH) and ms α Flotillin-1 (BD/610820). Secondary horseradish peroxidase (HRP)-coupled antibodies were obtained from Molecular Probes/Invitrogen.

Fusion constructs of α-syn-hGLuc1 (S1), α-syn-hGLuc2 (S2), [87, 88] as well as VPS4_WT and VPS4_DN constructs (kind gift of Prof. Dr. Nobuyuki Tanaka) [32, 89] have been previously described. GGA2-myc and GGA3-myc plasmids [79] were generated by cloning into pcDNA3.1(−) Myc/His A (Invitrogen) as previously described for GGA1-myc [57]. GGA3-mRFP was generated by cutting GGA3 cDNA out of the GGA3-myc construct using NheI and EcoRI cleavage site and subcloning into NheI and EcoRI sites of mRFP-N1 (Roger Tsien).

### Cell culture and transient transfection of N2A and HEK293

Human embryonal kidney cells (HEK293) (DSMZ no.: ACC 305) and murine neuroblastoma cells (Neuro-2a (N2A)) (DSMZ no.: ACC 148) were maintained in DMEM medium supplemented with 10% fetal bovine serum (both Invitrogen) and incubated at 37°C and 5% CO_2_. Cells were plated 24 hours prior to transfection, growing to 70-80% confluency prior to transfection. Media was changed to OptiMEM (Invitrogen) before transfection. Transient transfection was performed using Satisfection (Stratagene) (N2A) according to the manufacturer's instructions or calcium phosphate transfection method (HEK293). For conditioned media experiments, media was collected 48 hours post-transfection and centrifuged for 5 min at 3000g to eliminate floating cells prior to use.

### Protein knock-down

N2A cells were grown and handled as described above. Gga knock-down was performed by transfection of FlexiTube GeneSolution siRNA (Qiagen) against Gga1 (GS106039), Gga2 (GS74105) and Gga3 (GS260302) using Lipofectamine 2000 (Invitrogen) following the manufacturer's instructions. For the negative control, we used AllStars Neg. siRNA AF 488 (Qiagen, Cat.No: 1027292). Experiments were performed 48 h after transfection. Protein knock-down was evaluated by Western blot.

### Gaussia luciferase protein-fragment complemen-tation assay

HEK293 and N2A cells were seeded in 12 or 24 well plate formats as described above. Then, 48h after transient transfection with S1/S2 and additional expression constructs/siRNA, culture media was removed and centrifuged for 5 min at 3000g to eliminate floating cells. Then, 100μl was transferred to a white 96-well plate (Costar, Corning, NY, USA). Cells were washed with PBS and detached using Trypsin-EDTA (Invitrogen). Cells were resuspended in DMEM containing 10% FCS and centrifuged for 5 min at 500g. Cells were resuspended in OptiMEM and 100μl were transferred to a white 96 well plate. Luciferase activity from protein complementation was measured for conditioned media and live cells in an automated plate reader at 480 nm following the injection of a cell permeable substrate, coelenterazine (20 μM) (PJK), with a signal integration time of 2 seconds.

### Exosome isolation

Exosomes from N2A cells (1*10^6^/100 mm dish) were prepared as described previously [68, 69] with minor modifications. Briefly, conditioned medium of N2A cells (1*10^6^/100 mm dish) was collected 48h after transfection and spun for 5 min at 500 g to remove floating cells. The supernatants were then sequentially centrifuged at 300 g (10 min) and 2 x 200 g (10 min) at 4°C each. Then, supernatants were filtered through 0.45 μm (Whatmann, Florham Park, NJ) and then 0.22 μm (Millipore, Carrigtowhill, Cork, Ireland) filters, and centrifuged for 30 min at 10,000 g (2 x) at 4°C. After ultracentrifugation at 100,000 g for 70 min at 4°C, exosomal pellet was resuspended in BEX lysis buffer for Western blot analysis or OptiMEM for luciferase assay. Exosome depleted medium was prepared as described above, except after ultracentrifugation at 100,000 g for 70 min at 4°C, the exosome-free supernatant was filtered through a 0.22 μm filter before use. Purification was controlled by Western blot analysis using an antibody directed against Flotillin.

### Western blotting

Samples were lysed in BEX Lysis buffer (25 mM Tris pH 8.0, 20 mM NaCl, 0.6% w/v Deoxycholate, 0.6% Igepal CA-630) containing 1x HALT protease and phosphatase inhibitor cocktail (Pierce) and centrifuged for 15 min at 4°C and >13,000 g. Lysates were electrophoresed under denaturing conditions using NuPage Novex Bis-Tris 4-12% gradient gels and MES running buffer (both Invitrogen) according to the manufacturer's instructions. Proteins were transferred onto PVDF membranes (Roche) by wet blot using XCell II Blot Modules (Invitrogen). Membranes were blocked in 1x RotiBlock (Roth) for 1 h. Incubation with primary antibodies was performed overnight at 4°C in 1x RotiBlock. On the next day, after three 10 min washing steps in PBS-T, blots were incubated with HRP-coupled secondary antibodies for 1 h at room temperature. After three washing steps in PBS-T, blots were analyzed using the LAS-4000 CCD imager (GE Healthcare) and Luminata forte ECL (Millipore). Densitometric analysis was performed using ImageQuantTL (GE Healthcare).

### Toxicity assays

Toxicity was analyzed 48 h after transfection using the ToxiLight BioAssay Kit (Lonza, Rockland, ME), which quantitatively measures the release of adenylate kinase (AK) from damaged cells.

To investigate whether toxic properties of conditioned media from S1/S2 transfected N2A cells are altered upon GGA co-transfection, conditioned media was collected as described previously and added to naïve N2A cells seeded 24h before in 96-well plates. Media of naïve N2A cells was completely replaced by 100μl conditioned media from different transfection conditions. After incubation for 72 h, Caspase-Glo® 3/7 Assay was performed according to manufacturer's protocol (Promega, Madison, WI, USA).

For toxicity measurement of exosome and exosome-free fractions the conditioned media were treated as described above. Exosomes were resuspended in OptiMEM. Exosome and exosome-free fractions for toxicity assays were adjusted for luciferase counts and volume with OptiMEM prior to experiments to ensure that equal α-syn oligomer amounts were used. Both, exosome containing and exosome-free media were transferred to naïve N2A cells. After incubation for 72 h, Caspase-Glo® 3/7 Assay (Promega, Madison, WI, USA) was performed.

### Separation of monomeric and oligomeric α-syn using size exclusion chromatography

Size exclusion chromatography was performed as previously described [90]. Briefly, the Superdex 200 (10/300GL) column coupled to an Äkta Purifier (GE Healthcare) was equilibrated with PBS. Molecular mass was estimated according to manufacturer's instruction using Gel Filtration Calibration Kit (GE Healthcare) with standard samples: ferritin (440 kDa), aldolase (258kDa), conalbumin (75kDa), ovalbumin (44kDa) and carbonic anhydrase (29kDa). For the experiments, CM derived from HEK293 cells transfected with S1/S2 and GGA1, 2 3 or control was collected and centrifuged prior to use for 5 min at 20,000g. Then, 2 ml of CM was injected into the column and proteins were eluted with equilibration buffer (PBS) at a flow rate of 0.75 ml/min and the eluate was monitored at 215-280 nm. SEC fractions of 0.5 ml were collected and further analyzed for α-syn using luciferase assay and Dot Blots. For luciferase assay 100 μl eluate of each fraction were analyzed using the Gaussia luciferase protein-fragment complementation assay, as described above. For Dot Blot analysis, 300μl of each fraction was transferred onto nitrocellulose membranes (GE Healthcare) using the Bio-Dot Apparatus (Bio-Rad Laboratories, München, Germany). Membranes were blocked in 1x RotiBlock (Roth) for 1 h. Incubation with α-syn Antibody (BD/610787) was performed overnight at 4°C in 1x RotiBlock. On the next day, after three 10 min washing steps in PBS containing 0.1% Tween-20 (PBS-T), blots were incubated with HRP-coupled secondary antibodies for 1 h at room temperature. After three washing steps in PBS-T, blots were analyzed using the LAS-4000 CCD imager (GE Healthcare) and Luminata forte ECL (Millipore).

### Co-immunoprecipitation

HEK293 cells grown in 100 mm dishes were transiently co-transfected with alphα-synuclein constructs (S1+S2) and empty control vector or myc-tagged GGA3-wild type. Twenty-four hours after transfection, cells were washed with PBS and lysed in precooled lysis buffer (Miltenyi Biotec) on ice. Cell lysates were centrifuged for 15 min at 4°C and 16.000 rcf and supernatants were transferred to a fresh tube and incubated with anti-Myc-coated MicroBeads (Miltenyi Biotec) for 1 h on ice. Labelled Myc-tagged proteins were purified on MicroColumns (Miltenyi Biotec) following the manufacturer's instructions. Proteins were eluted with 50 μl preheated (95°C) NuPAGE lithium dodecyl sulfate (LDS) sample buffer (Invitrogen) containing 100 mM dithiothreitol and analyzed by LDS-PAGE and Western blotting.

### Human tissue

Human postmortem substantia nigra and cerebellum samples from Non-PD-controls and PD patients were provided by the University Hospital Ulm (Germany) in accordance with the law and permission of the local ethical committees. All experiments were performed in accordance with the Declaration of Helsinki. Written, informed consent to participate in this study was provided. For Western blot analysis, brain tissue was homogenized in BEX lysis buffer containing Halt phosphatase/protease inhibitor cocktail (Pierce) and 0.2% SDS.

### RNA extraction, cDNA synthesis, and quantitative PCR

Total RNA was extracted using the RNeasy Mini Plus Kit (Qiagen, Hamburg, Germany) in accordance with the manufacturer's instructions. Briefly, frozen tissues were placed into a tube containing a 5 mm stainless steel bead. Working on ice, 700μL of buffer RLT supplemented with 10% β-mercaptoethanol was added, and tissues were homogenized using a TissueLyser (Qiagen, Valencia, CA) at 30 Hz for 2 min. RNA concentrations were measured using a NanoDrop device (ThermoScientific, Waltham, MA). Then, 1 μg of total RNA was used to synthesize cDNA using iScript reverse transcriptase (Bio-Rad Laboratories, München, Germany) containing oligo-dT primers and random primers. cDNAs were diluted 10 times and PCR analysis was performed on a Bio-Rad iCycler System using iQSYBR Green Supermix (Bio-Rad Laboratories, München, Germany). A specific standard curve was performed in parallel for each gene to assess the specificity of the products, for quantification of the respective transcripts in duplicate. PCR conditions were 3 min at 94°C, followed by 40 cycles of 45 s at 94°C and 10 s at 60°C. The relative level of each RNA was normalized to two housekeeping genes (polymerase II and TBP). Oligonucleotide sequences for GGA3 quantification were 5′-ctgacgtacctgggggaca-3′ (fwd) and 5′cagactgcactatgccctgt-3′(rev).

### Statistical analysis

Statistical analyses were carried out using the program SigmaPlot13. Values in the figures are expressed as means +/− SD or +/− SEM.

## SUPPLEMENTARY MATERIAL FIGURES AND TABLE


