# Supplementary material for: The Golgi-localized, gamma ear-containing, ARF-binding (GGA) protein family alters alpha synuclein (α-syn) oligomerization and secretion
Source: Aging (Albany NY). 2017 Jul 15;9(7):1677–95. doi: 10.18632/aging.101261 (PMC5559169; doi:10.18632/aging.101261)
Supplement: Supplementary file 1 [file aging-09-1677-s001.pdf]

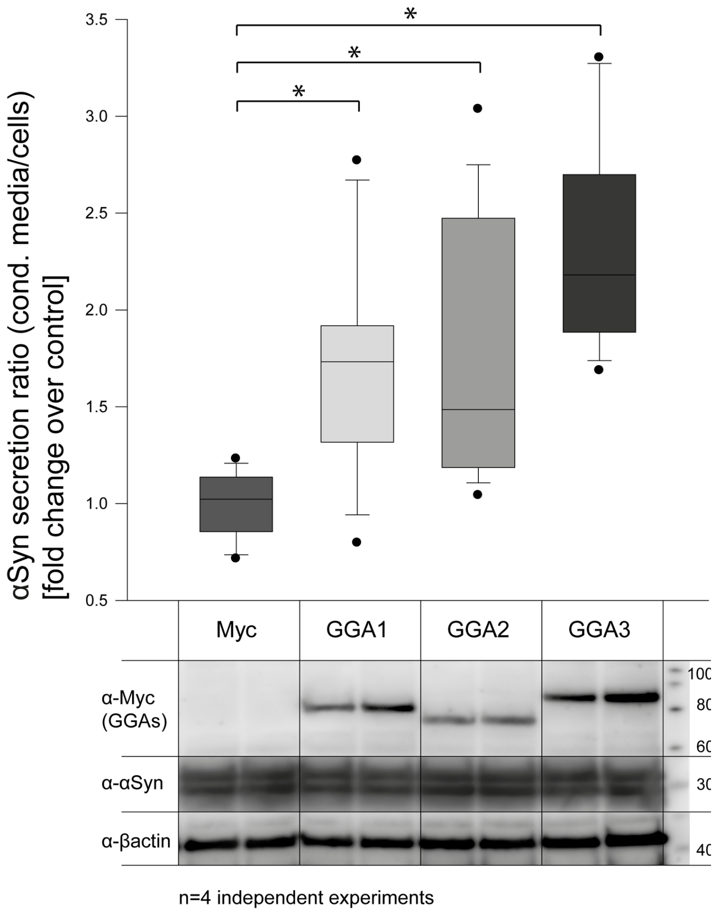

Figure S1. GGAs alter α-synuclein oligomer secretion in HEK293 cells. HEK293 cells were co-transfected with α-syn fused to non-bioluminescent N- or C-terminal fragments of *Gaussia princeps* luciferase (α-syn-hGLuc1 (S1) and α-syn-hGLuc2 (S2)) and either empty control or one member of the GGA protein family. Then, 48 h after transfection luciferase activity, indicative for α-syn oligomers, was determined in conditioned media as well as cells. The ratio of secreted α-syn oligomers in the conditioned medium to intracellular α-syn oligomers was built showing the increased secretion of α-syn oligomers for GGA1, GGA2 and GGA3. Experiments were carried out in triplicate; the results of n=4 independent experiments are shown. Statistical analysis was performed by using Kruskal-Wallis one-way ANOVA on ranks (*=p<0.05).

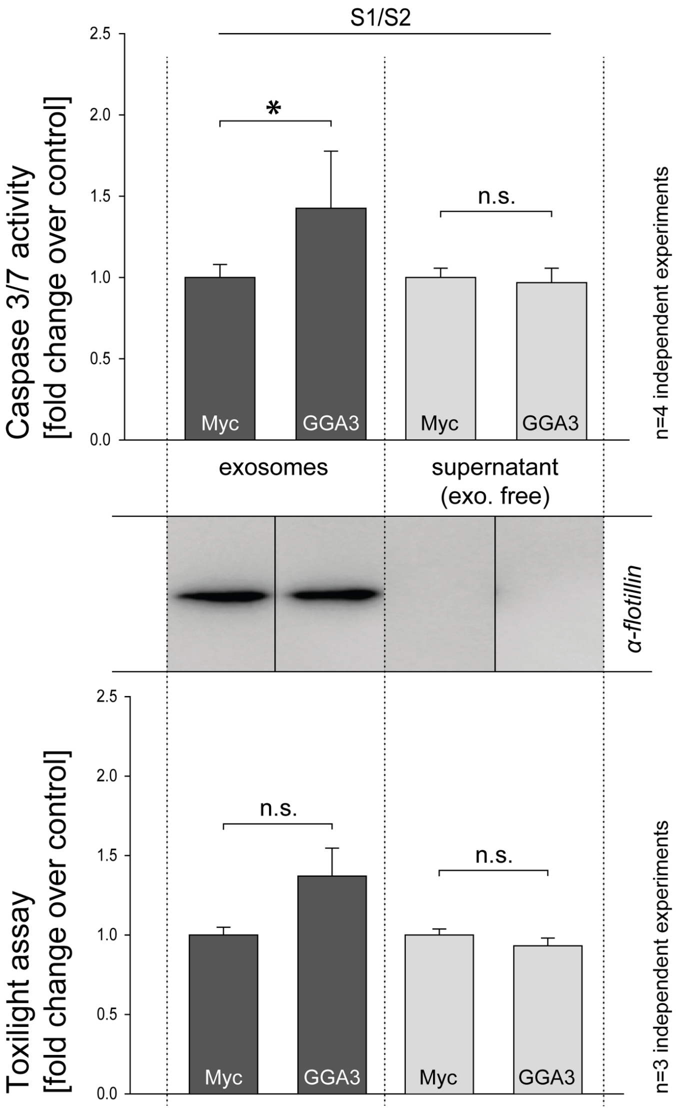

Figure S2. GGA3 enhances α-syn oligomer toxicity mainly in exosomes. Conditioned media from N2A cells co-expressing S1/S2 and GGA3 or myc control were collected 48h after transfection. Exosomes were isolated from CM by subcellular fractionation. Purity of the fractions was confirmed by Western blot and the exosome-specific marker flotilin. Exosomes were resuspended in OptiMEM. Equal volumes (100µl) of exosome-loaded OptiMEM and exosome-free OptiMEM were transferred to naïve N2A cells plated on a 96 well plate. After 72h, cells were analyzed for altered Caspase 3/7 activity (upper panel). We found increased toxicity of the conditioned media from GGA3 expressing cells to be mediated mainly by the exosome-loaded condition. In contrast, we found no difference in the toxicity of naïve N2A cells treated with exosome-free media. Experiments were carried out using 3-6 technical replicates; the results of n=4 independent experiments are shown. Additionally, conditioned media of the treated cells was analyzed by the ToxiLight assay (lower panel). An increase in toxicity could be observed in the supernatant of GGA3-exosome treated compared to myc-exosome treated cells. No increase was found in the exosome-free supernatants. Experiments were carried out using 3-6 technical replicates; the results of n=3 independent experiments are shown. Statistical analysis was performed by using Mann-Whitney Rank Sum Test (*=p<0.05).

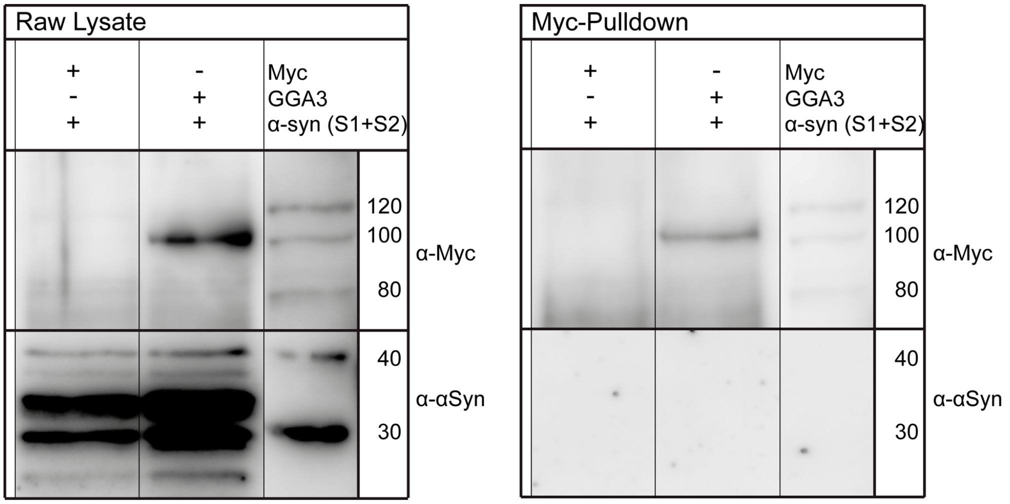

Figure S3. GGA3 does not interact with α-syn in HEK293 cells. HEK293 cells co-expressing S1/S2 and either GGA3 or myc control were lysed 24 h after transfection. Co-immunoprecipitation was performed using the anti-Myc MicroBeads (Miltenyi Biotech) following the manufacturer's instructions. Non-specific binding of α-syn to myc-tag or the column was excluded (left lane). α-syn was not co-precipitated by GGA3 (right lane) indicating that no direct interaction occurs between both proteins.

Table S1. Characteristics of human sample donors.

ID	gender	age	PMI [h]	co-morbidities	cause of death	PD Braak stage	Western blot	RT-qPCR
**PD #1**	m	77	24	dementia	myocardiac infarct	5	X	X
**PD #2**	m	79	48	depression, Binswanger's disease	embolism of the lung arteries	6	X	X
**PD #3**	m	82	84	no	broncopneumonia, sepsis	5		X
**PD #4**	m	71	18	dementia	broncopneumonia, sepsis	5		X
**PD #5**	m	75	18	dementia	broncopneumonia, sepsis	5		X
**PD #6**	m	76	8	dementia	coronary disease	5		X
**ID**	**gender**	**age**	**PMI [h]**	**co-morbidities**	**cause of death**	**PD Braak stage**	**Western blot**	**RT-qPCR**
**control #1**	m	81	12	no	N/K	N/A		X
**control #2**	f	91	6	prior minor stroke	N/K	N/A		X
**control #3**	f	73	72	no	N/K	N/A		X
**control #4**	m	92	8	Binswanger's disease	N/K	N/A		X
**control #5**	m	66	24	no	sepsis	N/A		X
**control #6**	m	45	24	no	myocardiac infarct	N/A	X	X
**control #7**	m	46	29	no	heart failure	N/A	X	X

The table summarizes the characteristics of the patients from which the post mortem tissue samples have been obtained. N/K= not known; N/A=not applicable, PMI=post mortem interval.
